# Evaluation of the comparative accuracy of the complement fixation test, Western blot and five enzyme-linked immunosorbent assays for serodiagnosis of glanders

**DOI:** 10.1371/journal.pone.0214963

**Published:** 2019-04-05

**Authors:** Mandy Carolina Elschner, Karine Laroucau, Harisankar Singha, Bhupendra Nath Tripathi, Muhammad Saqib, Ian Gardner, Sheetal Saini, Subodh Kumar, Hosny El-Adawy, Falk Melzer, Iahtasham Khan, Praveen Malik, Carola Sauter-Louis, Heinrich Neubauer

**Affiliations:** 1 Institute of Bacterial Infections and Zoonoses, Friedrich-Loeffler-Institut (FLI), Federal Research Institute for Animal Health, Jena, Germany; 2 Paris Est University, Animal Health Laboratory, EU-Reference Laboratory for Glanders, Maisons Alfort Cedex, France; 3 Indian Council of Agricultural Research—National Research Centre on Equines, Hisar, India; 4 Department of Clinical Medicine and Surgery, University of Agriculture Faisalabad, Faisalabad, Pakistan; 5 Department of Health Management, Atlantic Veterinary College, UPEI, Prince Edward Island, Canada; 6 Defence Research and Development Establishment, Microbiology Division, Gwalior, India; 7 Faculty of Veterinary Medicine, Kafrelsheikh University, Kafr El-Sheikh, Egypt; 8 Section of Epidemiology and Public Health, College of Veterinary and Animal Sciences, Jhang, Pakistan; 9 Chaudhary Charan Singh, National Institute of Animal Health, Department of Animal Husbandry, Dairying and Fisheries, Baghpat, India; 10 Institute of Epidemiology, Friedrich-Loeffler-Institut, Federal Research Institute for Animal Health, Greifswald-Insel Riems, Germany; Institut National de la Recherche Agronomique, FRANCE

## Abstract

Glanders is a zoonotic contagious disease of equids caused by *Burkholderia* (B.) *mallei*. Serodiagnosis of the disease is challenging because of false-positive and false-negative test results. The accuracy of the complement fixation test (CFT) which is prescribed for international trade by the World Organisation for Animal Health (OIE), five ELISAs and a Western blot (WB) were compared for serodiagnosis of glanders using sera from 3,000 glanders-free and 254 glanderous equids. Four ELISA tests are based on recombinant antigens (TssA, TssB, BimA and Hcp1), the IDVet ELISA is based on a semi-purified fraction of *B*. *mallei* and WB makes use of a purified LPS-containing *B*. *mallei*-antigen. Sensitivity and specificity of tests were estimated using cut-off values recommended by the test developers. The WB and all ELISAs, except BimA, were significantly more specific than the CFT. ELISAs based on TssA, TssB, and BimA antigens had significantly lower sensitivity compared to CFT while the sensitivities of the Hcp1-ELISA, the IDVet-ELISA and the WB did not differ significantly from that of the CFT. Given their comparable sensitivities and specificities, the CFT (98.0%, 96.4%), the WB (96.8%, 99.4%), the Hcp1-ELISA (95.3%, 99.6%) and the IDVet-ELISA (92.5%, 99.5%) should be further developed to meet OIE requirements.

## Introduction

Glanders is an infectious zoonotic disease of equids, caused by the gram-negative bacterium *Burkholderia* (*B*.) *mallei*. Although the disease has been eradicated in most European and North American countries in the last century, there are still outbreaks throughout the South American and Asian continents [1–5]. The disease is mainly chronic in horses and latently-infected animals pose a high risk of reintroduction of the infection into glanders-free countries [6]. Therefore, trade restrictions with animals and products from endemic regions or outbreak areas are mandatory.

Bacterial isolation and identification of *B*. *mallei* from cutaneous lesions and nasal exudates are considered to be the “gold” (perfect reference) standard for diagnosis of glanders. As approximately 90% of early infections occur in a non-clinical or latent form, clinical and bacteriological diagnosis are difficult [7].

Currently, mallein (allergic hypersensitivity test) and complement fixation test (CFT) are used for indirect diagnosis [8]. The CFT is the World Organisation for Animal Health (OIE) prescribed serodiagnostic method for international trade purposes and is also recommended for surveillance investigations [7]. This test is known to have high sensitivity, but it gives a considerable number of false-positive results [8, 9]. These false-positive results cause unnecessary restrictions on international trade of animals and products thereof and result in financial losses for owners and the horse industry. Test antigen used and the assay protocol have a significant impact on CFT sensitivity [10–12]. Antigens used for CFT are not standardized and they have different diagnostic accuracies. Moreover, the CFT is a labour intensive test and takes about 24h to perform. Recently, a CFT antigen based on a mixture of antigens from different historical serogroups has been extensively used [13]. Thus, the selection of antigens used for CFT has also to be updated and standardized [12]. Various immunodiagnostic tests such as indirect hemagglutination test, counter immunoelectrophoresis test, indirect fluorescent antibody test and enzyme-linked immunosorbent assays (ELISA) have also been previously used but have limitations [14].

Recently, new serological tests have been developed to overcome the disadvantages of the CFT i.e. complexity, anti-complementary reactions and poor standardizability [15]. Alternative methods such as the Western blot (WB) and ELISAs have been applied but have not been fully validated in large-scale studies [5, 9, 16–18] according to the principles and methods of validation of diagnostic assays for infectious diseases [19]. The Western blot, using a LPS-preparation, showed a markedly higher diagnostic specificity when compared to the CFT and was used as a confirmatory test for glanders [9].

ELISAs developed include a competitive ELISA with a high sensitivity and specificity that can be used in various host species [20]. Several purified recombinant proteins of *B*. *mallei* were also evaluated for use in ELISA, e.g. the intracellular motility A protein (BimA) [14], the type 6 secreted protein TssB [18] and a heat shock protein GroEL [21]. A new indirect ELISA based on a semi-purified fraction of *B*. *mallei* was recently developed [22].

In regions where *B*. *pseudomallei* occurs endemically, all currently-available serological diagnostic tests are unable to differentiate between *B*. *mallei* and *B*. *pseudomallei*, because of their close phylogenetic relationship.

However, to our knowledge, there has not been a comprehensive comparative evaluation study on these diagnostic tests using a suitable panel of well-characterized true-positive and true-negative field sera from different geographical origins. Therefore, this prospective study compared the diagnostic accuracy (sensitivity and specificity) of the WB technique, five indirect ELISAs (iELISA) and the OIE-prescribed CFT for the serological detection of *B*. *mallei* antibodies in equids. Data were analysed based on the assumption that the true infection status of animals was known with certainty and also in a Bayesian framework, where infection status was considered not known.

## Materials and methods

### Sera

#### Samples from glanderous animals

True positive serum samples (n = 254) were collected from equids in which the infection was confirmed by clinical signs (n = 112) or by *B*. *mallei* isolation or molecular detection of *B*. *mallei* by real-time PCR (n = 142) as recommended in the OIE manual [7]. The animals were considered to be “clinically positive” (n = 112) on the basis of the presence of at least one clinical sign consistent with glanders and the fact that they were detected during a culture-confirmed glanders outbreak in this population including close contact to infected animals. The 112 clinically positive samples were collected in 2016 and 2017 during glanders outbreaks in India (n = 111) and Pakistan (n = 1). The 142 culture or PCR positive samples were collected during glanders outbreaks in Pakistan in 2007 and 2016 (n = 124) and from India (n = 16) in 2016 and 2017. Two samples originated from horses testing positive in Germany in 2006 and 2014. The whole collection consisted of 226, 23 and 5 sera from horse, mule and donkeys, respectively. No further demographic data were available.

#### Samples from animals not infected with *B*. *mallei*

True negative samples were collected in countries officially free from glanders, to exclude exposure to *B*. *mallei* as much as possible. The risk that these animals were in contact with *B*. *pseudomallei* is negligible, because the regions of origin of the sampled animals do not belong to the known *B*. *pseudomallei* endemic regions [23]. In total, 3,000 true negative samples from three different geographical areas were used. The “Asia” batch (n = 1,000) included sera from Qatar, Saudi Arabia, Egypt, Bahrain, Jordan, UAE, Kuwait, and Oman, provided in 2015. The “South-America” batch (n = 1,000) was collected mostly from Buenos Aires, south of Santa Fe and south of Cordoba provinces in 2015. The “Europe” batch (n = 1,000) included samples from Germany and Ukraine was collected from 2011 to 2014. All samples from the “Asia” and “South-America” batches and most samples from Germany were unsystematically collected during routine testing for trade or movement. Some German samples were collected during equine infectious anemia surveillance. Samples from Ukraine were collected during a scientific surveillance study conducted on glanders. No further demographic data are available.

There were no adverse reactions recorded for blood collection. All serum samples were dispensed in 96 deep-well plates for each test and stored at -20°C to avoid multiple freezing-thawing cycles. Serological testing, as described in the following sections, was done independent of knowledge of infection status of the donor.

### Ethics statement

For this study no ethical approvals were required. All blood samples were routinely collected for prescribed diagnostic purposes or official monitoring studies and subsequently made available to the study.

### Description of the tests

The characteristics of the tests compared in this study are given in Table 1.

**Table 1:** Name of the test, test developer and used antigens of the 7 compared assays.

Test	Developer/ protocol used	Antigen used	Abbreviation
Complement fixation test	OIE-Manual [7]	Malleus-CFT antigen; Ccpro GmbH, prepared from *B*. *mallei* strain Ivan-NCTC 10230	CFT
Western blot	FLI, Germany [9]	LPS containing antigen consisting of 3 different *B*. *mallei* strains (Bogor, Mukteswar, Bahrain1)	WB
ID Screen Glanders Indirect ELISA	EU-Reference laboratory/ ID.Vet, France	Semi-purified antigen fraction prepared from *B*. *mallei* strain ATCC 23344	IDVet
Indirect TssA ELISA	ICAR-NRCE, India	Recombinant protein TssA of Type 6 secretory system of *B*. *mallei* (Type strain)	TssA
Indirect TssB ELISA	ICAR-NRCE, India [18]	Recombinant protein TssB of Type 6 secretory system of *B*. *mallei* (Type strain)	TssB
Indirect Hcp1 ELISA	ICAR-NRCE, India	Recombinant protein Hcp1 of Type 6 secretory system of *B*. *mallei* (Type strain)	Hcp1
Indirect BimA ELISA	ICAR-NRCE, India [14]	Recombinant protein BimA of *B*. *mallei* (Type strain)	BimA

#### Complement fixation test (CFT)

CFT was performed as described in the OIE manual [7]. Briefly, serum samples were diluted 1:5 in CFT buffer (Institute Virion/ Serion GmbH), inactivated and two fold dilutions of them were mixed with Malleus CFT antigen (Ccpro GmbH) and 5 complement haemolytic units-50% of guinea pig complement (Institute Virion/ Serion GmbH). Sera, complement and antigen were mixed in the plates and incubated overnight at 4°C. A 2% suspension of sensitized (amboceptor from Institute Virion/ Serion GmbH) sheep red blood cells (Labor Dr. Merk & Kollegen) were added and plates were incubated for 45 minutes at 37°C and then centrifuged for 5 minutes at 600 g. Samples with 100% hemolysis in a dilution of 1:5 were categorized as negative, samples showing 25–75% hemolysis in a dilution of 1:5 were classified as suspicious and samples showing 100% inhibition of hemolysis in a dilution of 1:5 were classified as positive. All suspicious test results were classified as positive.

#### Western blot (WB)

The antigen preparation and WB were performed as described previously [9]. In the aforementioned study on the establishment of Western blot, the *B*. *mallei* isolates Bogor, Mukteshwar and Zagreb, known for several decades, were used. For the study described here, the strain Zagreb was replaced by a more recent isolate from Bahrain. Briefly, the *B*. *mallei* strains Bogor (originating from Indonesia), Mukteswar (originating from India) and Bahrain1 (isolated from a horse from Bahrain in 2011) were cultivated on blood agar plates. For LPS purification, *B*. *mallei* colonies were re-suspended thoroughly in 6 ml saline (0.9% NaCl, pH 7.0) up to a density comparable to McFarland scale 4.0. The cells were treated with formaldehyde in a final concentration of 12.3% by overnight shaking. The cells were pelletized by centrifugation at 3,500g for 15 min and washed 3 times using PBS. The formaldehyde treatment and the subsequent 3 washings were repeated twice. After the last washing step the pellet was resuspended in PBS and the correct formation of a typical LPS ladder was shown by SDS-PAGE and silver staining before blotting. The batches of the three antigens were made available in a lyophilized form. For the immunoblot analysis the antigens were mixed equally and blotted onto a 0.45 μm nitrocellulose membrane (Invitrogen, Germany). Five millimeter strips of the membrane were used to analyze the serum samples at a dilution of 1:50. Anti-horse conjugate labelled with alkaline phosphatase (Sigma, Munich, Germany) was used in a dilution of 1:5,000. Detection was made by the ready-to-use NBT-BCIP staining (Sigma). The WB was scored positive, if the banding pattern of the LPS ladder was clearly visible, scored suspicious if a reaction was seen within the region of 20 to 60 kilodalton (kDa), and scored negative if no reactions were seen in the area between 20 and 60 kDa. All suspicious (intermediate) test results were classified as positive.

#### Recombinant BimA-, Hcp1-, TssA- and TssB-ELISA

The four recombinant ELISAs were performed according to the developer’s instructions. Expression and purification of recombinant BimA and TssB antigens were described previously [14, 18], codon optimized and synthetic gene sequences of *B*. *mallei* loci BMAA0742 and BMAA0744, respectively, were expressed in pQE30 vector (Qiagen). Recombinant Hcp1 and TssA antigens were purified by Ni^+^-NTA agarose (Qiagen) column chromatography according to manufacturer’s instruction. Optimization of ELISA protocol and diagnostic cut offs were determined using a panel of glanders positive and negative sera (unpublished data).

Briefly, the ELISA plates (Maxisorp, Nunc) were coated with 100 μl of the antigen diluted in coating buffer (Sigma-Aldrich) in a concentration of 75 ng/well (Hcp1, BimA) or 150 ng/well (TssA, TssB) and incubated at 4°C overnight. On the second day, the plates were washed three times (each 200 μl/well) using washing buffer (PBS with 0,05% Tween-20 (Sigma-Aldrich)), blocked for one hour at 37°C using 100 μl/ well blocking buffer (washing buffer with 6% skim milk powder, Merck-Millipore) and washed five times. The serum samples were tested in duplicates pre-diluted 1:200 in dilution buffer (PBS with 3% skim milk powder, 100 μl/well) and after incubation of 1 hour at 37°C washed 5 times. Subsequently, the anti-horse horse radish peroxidase (HRPO) conjugate (Sigma-Aldrich) diluted 1:10,000 in dilution buffer was added (100 μl/well) and the plates were incubated at 37° for one hour. After five washing steps, each well was filled with 50 μl substrate solution (Ready to use 1-Step ultra TMB ELISA substrate; Thermo Scientific). After processing in a dark place (Hcp1 and BimA for 5 minutes, TssA and TssB for 10 minutes), the reaction was stopped by addition of 1M sulfuric acid solution (Carl Roth) OD 450 nm was recorded. Sample/Positive ratio (S/P%) was calculated for each sample (OD Sample—OD Negative Control) / (OD Positive Control–OD Negative Control) x 100. Sera with S/P% values ≥ 10 (BimA), ≥ 15 (TssA) and ≥ 20 (TssB, Hcp1) were considered as positive.

#### ID Screen Glanders ELISA (IDVet)

The IDVet test (ID Screen Glanders ELISA, IDVet, Grabels, France) was provided as a ready-to-use kit and was performed according to the manufacturer’s instructions. The kit contained *B*. *mallei* antigen-coated plates with individual 8-well strips, positive and negative controls, 10x concentrated multi-species horseradish conjugate, dilution buffer, 20x wash concentrate, TMB substrate solution and stop solution. Briefly, 200 μl of the serum samples in a pre-dilution of 1:20 were added to an antigen-coated and non-coated well, respectively. After 45 ± 5 min incubation at 21 ± 5°C, wells were emptied, washed 3 times and incubated with the conjugate for 30 ± 3 min at 21 ± 5°C. Washing was repeated 3 times. Then the substrate was added and after incubation for 15 ± 2 min reaction was stopped and OD 450 nm was recorded. S/P% values were calculated and samples with S/P% ≤ 40% were considered as negative, between 40 and 50% as suspicious and ≥ 50% as positive. All suspicious (intermediate) test results were classified as positive.

### Statistical analysis

Ninety-five percent confidence intervals (CI) for sensitivity, specificity and likelihood ratios were based on standard formulas [24] and done using MedCalc version 13.1.0.0 (https://www.medcalc.org/).

Sensitivity and specificity covariances for pairs of tests (a measure of conditional dependence or correlation) were calculated as described in Gardner and others [25] using an Excel template and assuming the true infection status was known with certainty, as described in the sample description paragraph. Results were expressed as the percentage of maximum value and the obtained value is similar to a correlation coefficient. To account for the possibility of misclassification of the true infection status, data were reanalyzed in a Bayesian framework that accounted for uncertainty about true status and incorporated necessary covariance terms in a latent class model (LCM). The model was based on 4 tests (CFT, WB and the two most promising ELISAs) in two populations (infected and non-infected) and used code adapted from Branscum and others [26]. The model initially assumed conditional independence of tests, which is reasonable if the true status is known with certainty, constant sensitivity and specificity in both populations, and distinct prevalences. Flat priors (beta 1,1) were used for the sensitivity and specificity of all tests. Prevalence of infection in true-positive cases was assumed to have a mode of 100% with 95% probability that the true value was greater than 98% (modelled as a beta 148.3,1) and prevalence in the true-negative cases (pooled across the 3 regions) was assumed to have a mode of 0% with 95% probability that the true value was less than 2% (modelled as beta 1,148.3). Two sensitivity covariance terms were added to the model to allow for conditional dependence that was evident in the sensitivity data. Models were run in OpenBUGS version 3.2.3 [27] and model code is available from the authors on request. Median and 95% probability intervals were used for posterior inferences about sensitivity and specificity.

## Results

The frequency of combinations of the 4 serologic test results (positive/negative) in infected and non-infected populations is shown in Table 2.

**Table 2:** Frequency of combinations of test results in known infected and non-infected populations. Combinations of test results with zero counts are not listed in the table.

Infected population (PCR or culture positive)	n	Infected population (Clinical signs)	n	Non-infected population	n
CFT+ WB+ IDVet+ Hcp1+	123	CFT+ WB+ IDVet+ Hcp1+	100	CFT+ WB+ IDVet- Hcp1-	2
CFT+ WB+ IDVet+ Hcp1-	5	CFT+ WB+ IDVet- Hcp1+	7	CFT+ WB- IDVet+ Hcp1-	2
CFT+ WB+ IDVet- Hcp1+	4	CFT+ WB- IDVet+ Hcp1+	2	CFT+ WB- IDVet- Hcp1-	104
CFT+ WB+ IDVet- Hcp1-	3	CFT- WB+ IDVet+ Hcp1+	1	CFT- WB+ IDVet- Hcp1-	16
CFT+ WB- IDVet+ Hcp1+	1	CFT- WB+ IDVet- Hcp1+	1	CFT- WB- IDVet+ Hcp1+	1
CFT+ WB- IDVet- Hcp1+	1	CFT- WB- IDVet+ Hcp1+	1	CFT- WB- IDVet+ Hcp1-	11
CFT+ WB- IDVet- Hcp1-	3			CFT- WB- IDVet- Hcp1+	12
CFT- WB+ IDVet+ Hcp1+	1			CFT- WB- IDVet- Hcp1-	2811
CFT- WB+ IDVet+ Hcp1-	1				
Total	142	Total	112	Total	2959

### Diagnostic sensitivity (DSe) and diagnostic specificity (DSp)

The DSe was calculated for each test based on the results of 254 true-positive sera. Samples showing suspicious results were considered positive for calculation of point estimates and 95% CI for DSe. Firstly, both subgroups of 142 culture or PCR positive samples and 112 positives samples from animals only identified by clinical signs of glanders were assessed separately (Table 3). For evaluation of DSp, serum samples from glanders-free areas were used. Of the 3,000 tested samples, 41 showed anti-complementary activity. Excluding the results of these 41 samples for DSp calculations had no significant impact on DSp values (S1 Table). The separate analysis of 2,959 samples for DSp of the three different sample batches “Asia”, “South-America, and “Europe” did not show significant differences (S2 Table). Table 3 summarizes the results of all batches together.

**Table 3:** Diagnostic sensitivity (DSe) of 142 culture or PCR positive proven samples, 112 positives samples from animals with clinical signs of glanders and of all 254 samples together, and diagnostic specificity (DSe) of 2,959 tested samples including the respective 95% CI.

DSe testing of 142 culture or PCR positive proven samples				
Assay	FN	TP	DSe%	CI 95%
CFT	2	140	98.6	95.0–99.8
WB	5	137	96.5	92.0–98.9
IdVet	11	131	92.2	86.6–96.1
Hcp1	12	130	91.6	85.7–95.6
BimA	23	119	83.8	76.7–89.4
TssA	21	121	85.2	78.3–90.6
TssB	28	114	80.3	72.8–86.5
DSe testing of 112 positives samples from animals with clinical signs of glanders				
Hcp1	0	112	100	96.8–100
CFT	3	109	97.3	92.3–99.4
WB	3	109	97.3	92.4–99.4
IdVet	8	104	92.8	86.4–96.9
BimA	14	98	87.5	79.9–93.0
TssA	22	90	80.4	71.8–87.3
TssB	15	97	86.6	78.9–92.3
DSe testing of all 254 positive samples				
CFT	5	249	98.0	95.5–99.4
WB	8	246	96.8	93.9–98.6
Hcp1	12	242	95.3	91.9–97.5
IdVet	19	235	92.5	88.6–95.4
BimA	37	217	85.4	80.5–89.5
TssA	43	211	83.1	77.9–87.5
TssB	43	211	83.1	77.9–87.5
DSp testing of 2,959 negative samples				
Assay	FP	TN	DSp%	
TssB	0	2,959	100	99.9–100
Hcp1	13	2,946	99.6	99.2–99.7
IdVet	14	2,945	99.5	99.2–99.7
WB	18	2,941	99.4	99.0–99.6
TssA	30	2,929	99.0	98.6–99.3
BimA	76	2,883	97.4	96.8–97.9
CFT	108	2,851	96.4	95.6–97.0

TN-true negatives, TP-true positives, FN-false negatives, FP-false positives

The CFT was the most sensitive test in this study (98.0%). The differences in DSe between CFT, IDVet, TssA, TssB and BimA were statistically significant (P<0.05), whereas there was no statistical evidence of differences in DSe between CFT, WB and Hcp1-ELISA (p>0.05). All pairwise comparisons of DSp were significantly different (p<0.05) except for WB-IDVET, WB-Hcp1, and WB-TssA (S3 Table).

### Combinations of tests

The highest sensitivity of pairs of tests in parallel (99.6%) was achieved by use of CFT and any of the other 3 tests (WB, IDVet- or Hcp1-ELISA). The remaining 3 combinations all had combined Se <99%. Use of test pairs of in series yielded values of 99.9% or 100% for all 6 combinations. Positive sensitivity and specificity covariances between tests decrease the gain in sensitivity for use of 2 tests in parallel (only one test results needs to be positive) and gain in specificity when 2 tests are used for serial interpretation (both test results need to be negative), respectively.

### Bayesian estimates of DSe and DSp

Estimates obtained in the Bayesian analysis were similar to those obtained in the traditional frequentist analysis based on known infection status (Table 4).

**Table 4:** Posterior median and 95% probability intervals for sensitivity (Se) and specificity (Sp) of the CFT, Hcp1, IDVet and WB tests for glanders in equids. Median is preferred to mean given the asymmetric distribution.

	Sensitivity			Specificity		
Assay	2.5th %	Median	97.5th%	2.5th %	Median	97.5th%
Subset of PCR and culture positive horses (n = 142)						
CFT	94.9	98.1	99.6	95.5	96.2	96.9
Hcp1	87.6	92.8	96.4	99.3	99.5	99.7
IDVet	88.6	93.5	96.9	99.2	99.5	99.7
WB	94.3	97.8	99.5	99.0	99.4	99.6
Subset of clinical cases (n = 112)						
CFT	92.4	96.0	99.0	95.6	96.3	97.0
Hcp1	96.7	99.4	99.9	99.3	99.5	99.7
IDVet	86.5	92.4	96.3	99.2	99.5	99.7
WB	92.4	96.7	99.0	99.0	99.4	99.6
All 254 horses						
CFT	95.4	97.7	99.1	95.5	96.2	96.9
Hcp1	93.1	96.0	98.0	99.3	99.5	99.7
IDVet	89.6	93.2	95.9	99.2	99.5	99.7
WB	95.0	97.6	99.1	99.0	99.4	99.6

### Likelihood ratios and predictive values

Table 5 shows the positive and negative likelihood ratio values of the four most promising tests (CFT, WB, IDVet and Hcp1-ELISA) as well as the positive and negative predictive values for scenarios of 0.1% and 3% prevalence, which were considered to be typical and worst cases for prevalence in equids. Based on the assumption that the true infection of equids was known, the prevalence in the study sample was 7.9% (254/3,213) which is substantially above estimates in naturally-infected populations.

**Table 5:** Likelihood ratios for CFT, WB, IDVet, Hcp1 and predictive values in scenarios of 0.1% and 3% disease prevalence.

Assay	CFT	CI 95%	WB	CI 95%	IDVet	CI 95%	Hcp1	CI 95%
LR+	26.9	22.3–32.4	159.2	100.4–252.5	195.6	115.8–330.2	216.9	126.0–373.3
LR-	0.02	0.01–0.05	0.03	0.02–0.06	0.08	0.05–0.11	0.05	0.03–0.08
Prevalence 0.1%								
PPV	2.6	0.6–7.3	13.8	3.0–35.0	16.4	3.4–41.1	17.8	3.9–43.6
NPV	100.0	99.9–100	100	99.9–100	100	99.9–100	100	99.9–100
Prevalence 3%								
PPV	45.4	38.5–52.4	83.2	74.9–89.5	85.8	77.6–91.9	87.0	79.1–92.8
NPV	99.9	99.8–100	99.9	99.7–100	99.8	99.5–99.9	99.8	99.6–100

LR+: Positive Likelihood Ratio; LR-: Negative Likelihood Ratio; PPV: Positive Predictive Value; NPV: Negative Predictive Value

## Discussion

In the present study, five ELISAs, a WB and the CFT were comparatively evaluated for the detection of antibodies against *B*. *mallei* in equid serum. The diagnostic accuracy of the CFT was analyzed following the guidance in the OIE manual [7]. The specificity of tests depends on cross-reacting organisms which can vary from biotope to biotope [28]. Therefore, serum samples were analyzed separately from 3 different geographical regions (“South-America” batch, “Europe” batch, “Asia” batch), but no significant differences in specificity were found. Based on all 3,000-tested serum samples in this study, it was shown, that the five ELISAs and the WB were more specific than the CFT. Previously published reports showed the utility of ELISA techniques regarding the improvement of specificity in comparison to CFT. The specificity of ELISA using either recombinant BimA antigen or TssB protein were 98.9% and 100%, respectively [14, 18]. The WB was previously shown to have a specificity of 100% [9]. Another study showed the IDVet-ELISA to have excellent specificity (98.9%) in a preliminary validation [22]. However, the estimates were based on lower sample numbers than considered adequate by OIE.

The evaluation of test sensitivity showed the best results for the CFT followed by WB, Hcp1-, and IDVet-ELISAs. Because of the outstanding sensitivity of the CFT, alternative methods must be benchmarked against it. Hence, OIE requires a confirmatory test for glanders with equal or higher sensitivity and higher specificity [7]. Especially in areas where the number of false positives is very high, the lack of specificity of the CFT results in constant obstacles to trade in equidae and poses a constant challenge for veterinary authorities [6]. In international trade, the most sensitive test for highly transmissible and pathogenic agents has to be applied to avoid an introduction of infected animals into disease free populations [29].

The biggest challenge in validation of serological tests for glanders is the lack of availability of a sufficient number of true-positive serum samples to statistically compare sensitivity across multiple tests with high confidence. Positive sera, with positive isolation of *B*. *mallei*, are extremely difficult to collect. The horses tested positive are considered to pose a zoonotic risk or a source for future spread of the disease in the equine population and are destroyed immediately before samples can be collected. The areas of endemicity are often in remote regions and cooling chains cannot be maintained. In various countries serum collection is restricted by regulations and laws. Our study therefore had to use a hybrid design in which cases were selected based on clinical presentation and positive PCR or culture results. Non-infected samples were collected from countries officially free of infection. This design may have led to selection bias in estimates as glanders-positive cases may have been the most severely affected in source populations [30]. However, because sample inclusion was not based on positive results by any serologic tests, we believe this bias was not substantial and was unlikely to impact the sensitivity and specificity rankings of the tests. This study showed that a serum panel well characterized by confirmative clinical observations of experienced veterinarians could be useful to increase case sample numbers and thus increase statistical certainty.

Considering both sensitivity and specificity, the WB (96.8%, 99.4%), the Hcp1-ELISA (95.3%, 99.6%) and the IDVet-ELISA (92.5%, 99.5%) should be further developed to meet OIE demands in the near future. These three tests were available for the study in very different stages of development. The WB, as performed in the OIE reference laboratory at the FLI, was based on a semi-purified antigen and reading of WB results needs experienced operators. The in-house production of this test is expensive and thus this test is not appropriate for screening large numbers of samples in surveillance programs. Hence, it is suitable as a confirmatory test. The Hcp1-ELISA was available as a ‘semi-ready’ non-commercialized format. The ELISA plates had to be coated with antigens, blocked and washed before the test could be used. If this ELISA would be produced commercially, this test could be a promising tool for the future. The IDVet-ELISA was provided as a ready-to-use commercialized kit. The cut offs for the ELISAs and the WB were based on those recommended by the developer or manufacturer of the tests. It is obvious that other results will be obtained with different cut off values. Whether a cut off value shift is sufficient for the needs of OIE to improve the test properties of the ELISAs has to be proven by testing more positive samples and under consideration of the important prevalence-independent test characteristics of LR- and LR+.

In Germany, the method used to exclude false-positive CFT results is to run a WB as a confirmatory test on CFT-positive sera [31]. The results from the present study confirm that use of a combination of tests can enhance the accuracy of serological diagnosis.

However, taking into account the performance characteristics of assays based on the pre-determined cut off values used in this study, replacement of the CFT with the WB or any of the ELISAs cannot be recommended for testing equids for trade yet.

Presently, the CFT for glanders is still the prescribed technique for serological investigation of equids for trade purposes [7] to certify individual animal freedom from disease. The test is difficult to standardize, as there are no standardized sera available [12]. Furthermore, the CFT requires experienced operators, ongoing training and quality management systems to be implemented in the laboratory. The performance of the test is demanding and the results depend on the antigen used and methods, such as incubation conditions [10, 11]. Therefore, efforts to further improve and optimize the WB and ELISAs, e.g. combination of antigens, should be continued.

- Ullrich Wernery, Central Veterinary Research Institute, Dubai, United Arab Emirates
- Teótimo Becú, Clinica equina SRL, Laboratorio de Diagnóstico, Laboratorio de Producción de Biológicos, Buenos Aires, Argentina
- Roman Koziy, State Scientific Control Institute of Biotechnology and Strains of Microorganisms, Kiev, Ukraine

## Supporting information

S1 Table DSp values with and without 41 anti-complementary sera. (DOCX)

S2 Table Separate analysis of 2,959 samples for DSp of the three different sample batches “Asia”, “South-America, and “Europe”. (DOCX)

S3 Table Significance (p values) of differences in DSp for test pairs based on McNemar’s test for correlated proportions. (DOCX)
