# Supplementary material for: Evaluation of the comparative accuracy of the complement fixation test, Western blot and five enzyme-linked immunosorbent assays for serodiagnosis of glanders
Source: PLoS One. 2019 Apr 5;14(4):e0214963. doi: 10.1371/journal.pone.0214963 (PMC6450644; doi:10.1371/journal.pone.0214963)
Supplement: S1 Table — (DOCX) [file pone.0214963.s001.docx]

**S1 Table. DSp values with and without 41 anti-complementary sera.**

| Assay | N | FP | TN | DSp % | CI 95% |
| --- | --- | --- | --- | --- | --- |
| CFT | 2,959 | 108 | 2,851 | 96.4 | 95.6-97.0 |
| WB | 3,000 | 18 | 2,982 | 99.4 | 99.0-99.6 |
|  | 2,959 | 18 | 2,941 | 99.4 | 99.0-99.6 |
| Idvet | 3,000 | 14 | 2,986 | 99.5 | 99.2-99.7 |
|  | 2,959 | 14 | 2,945 | 99.5 | 99.2-99.7 |
| HCP1 | 3,000 | 13 | 2,987 | 99.6 | 99.3-99.8 |
|  | 2,959 | 13 | 2,946 | 99.3 | 99.2-99.7 |
| BimA | 3,000 | 76 | 2,924 | 97.5 | 96.8-98.0 |
|  | 2,959 | 76 | 2,883 | 97.4 | 96.8-97.9 |
| TssA | 3,000 | 30 | 2,970 | 99.0 | 98.6-99.3 |
|  | 2,959 | 30 | 2,929 | 99.0 | 98.6-99.3 |
| TssB | 3,000 | 1 | 2,999 | 100 | 99.8-100 |
|  | 2,959 | 0 | 2,959 | 100 | 99.9-100 |

FP-false positives, TN-true negatives
