# Supplementary material for: Evaluation of the comparative accuracy of the complement fixation test, Western blot and five enzyme-linked immunosorbent assays for serodiagnosis of glanders
Source: PLoS One. 2019 Apr 5;14(4):e0214963. doi: 10.1371/journal.pone.0214963 (PMC6450644; doi:10.1371/journal.pone.0214963)
Supplement: S2 Table — (DOCX) [file pone.0214963.s002.docx]

**S2 Table. Separate analysis of 2,959 samples for DSp of the three different sample batches “Asia”, “South-America, and “Europe”.**

| DSp testing of 984 negative samples from “Asia” | | | | |
| --- | --- | --- | --- | --- |
| Assay | FP | TN | DSp% | CI 95% |
| CFT | 38 | 946 | 96.14 | 94.74-97.17 |
| WB | 4 | 980 | 99.59 | 98.96-99.84 |
| IdVet | 2 | 982 | 99.80 | 99.26-99.94 |
| HCP1 | 5 | 979 | 99.49 | 98.82-99.78 |
| BimA | 27 | 957 | 97.26 | 96.04-98.11 |
| TssA | 22 | 962 | 97.76 | 96.6-98.52 |
| TssB | 0 | 984 | 100.00 | 99.61-100 |
| DSp testing of 980 negative samples from “South America” | | | | |
| Assay | FP | TN | DSp% |  |
| CFT | 8 | 972 | 99.18 | 98.4-99.59 |
| WB | 8 | 972 | 99.18 | 98.4-99.59 |
| IdVet | 3 | 977 | 99.69 | 99.1-99.9 |
| HCP1 | 26 | 954 | 97.35 | 96.14-98.18 |
| BimA | 4 | 976 | 99.59 | 98.96-99.84 |
| TssA | 0 | 980 | 100.00 | 98.08-100 |
| TssB | 27 | 953 | 97.24 | 96.02-98.1 |
| DSp testing of 995 negative samples from “Europe” | | | | |
| Assay | FP | TN | DSp% |  |
| CFT | 43 | 952 | 95.68 | 94.23-96.78 |
| WB | 6 | 989 | 99.40 | 98.69-99.72 |
| IdVet | 4 | 991 | 99.60 | 98.97-99.84 |
| HCP1 | 5 | 990 | 99.50 | 98.83-99.79 |
| BimA | 23 | 972 | 97.69 | 96.56-98.45 |
| TssA | 4 | 991 | 99.60 | 98.97-99.84 |
| TssB | 0 | 995 | 100.00 | 99.62-100 |

FP-false positives, TN-true negatives
