# Supplementary material for: Evaluation of the comparative accuracy of the complement fixation test, Western blot and five enzyme-linked immunosorbent assays for serodiagnosis of glanders
Source: PLoS One. 2019 Apr 5;14(4):e0214963. doi: 10.1371/journal.pone.0214963 (PMC6450644; doi:10.1371/journal.pone.0214963)
Supplement: S3 Table — (DOCX) [file pone.0214963.s003.docx]

**S3 Table. Significance (P values) of differences in DSp for test pairs based on McNemar’s test for correlated proportions.**

| Test | CFT | WB | IDVet | Hcp1 | BimA | TssA | TssB |
| --- | --- | --- | --- | --- | --- | --- | --- |
| CFT |  | < 0,0001 | < 0,0001 | < 0,0001 | 0,0216 | < 0,0001 | < 0,0001 |
| WB |  |  | 0,5959 | 0,4725 | < 0,0001 | 0,0865 | < 0,0001 |
| IDVet |  |  |  | 1 | < 0,0001 | 0,0171 | 0,0001 |
| Hcp1 |  |  |  |  | < 0,0001 | 0,0104 | 0,0002 |
| BimA |  |  |  |  |  | < 0,0001 | < 0,0001 |
| TssA |  |  |  |  |  |  | < 0,0001 |
| TssB |  |  |  |  |  |  |  |
